# Healthcare professionals’ knowledge of and compliance with the ASCO/ESMO/GLIM guidelines for the diagnosis and management of cancer cachexia (CC): the ASSIST-CC baseline findings in Uganda

**DOI:** 10.3332/ecancer.2024.1712

**Published:** 2024-06-07

**Authors:** Innocent Atuhe, Alfred Jatho, Babra Nalwadda, Judith Asasira, Martha Nantayi, Joseph Semujju, Naome Namwira, Kulusum Namayanja, Ashley Atwine, Semei Buwambaza Sekitene, Jackson Orem

**Affiliations:** 1 King Ceasar University, PO Box 88, Kampala, Uganda; 2 Newton & Einstein Institute of Science and Technology, PO Box 1330, Mbarara, Uganda; 3 Uganda Cancer Institute, PO Box 3935, Kampala, Uganda; 4 Mbarara Regional Cancer Centre, PO Box 3935, Kampala, Uganda

**Keywords:** cancer cachexia, cancer cachexia diagnosis, cancer cachexia management

## Abstract

**Background:**

More than 50% of people with advanced cancer suffer from cancer-related cachexia (CC) – a major contributor to morbidity and mortality. Despite the lack of local guidelines on CC diagnosis and management in Uganda, the American Society of Clinical Oncology (ASCO), the European Society for Medical Oncology (ESMO) and the Global Leadership Initiative on Malnutrition (GLIM) developed guidelines on CC screening and management. However, the level of knowledge on CC and compliance with the available guidelines among Ugandan oncology health professionals is unknown. This study aimed to assess the level of *awareness and knowledge of CC diagnosis and management and compliance with the ASCO/ESMO/GLIM guidelines on CC *among *healthcare professionals *(HCPs) involved in the care of cancer patients.

**Methods:**

In this phase one, a self-administered structured questionnaire developed using the ASCO/ESMO and GLIM guidelines on diagnosis and management of CC was used to assess the level of awareness, and knowledge of 200 health professionals from three hospital settings on CC, and compliance with the ASCO/ESMO/GLIM guidelines on CC related core communication, barriers to communication, clinician training in communication, discussing goals of care, treatment options and meeting the needs of the underserved populations. The data were entered into Research Electronic Data Capture software analysed using STATA version 18.0 software.

**Results:**

The overall objectively correct knowledge score of CC diagnosis criteria was 67.5% (*n* = 135), yet there was a much lower level of awareness about ASCO/ESMO/GLIM guidelines on CC at 30% (*n* = 60) and only 21% (*n* = 42) of the HCPs have ever assessed Quality of life of CC patients. The compliance with ASCO/ESMO/GLIM guidelines on nutritional interventions for patients with CC varied across the variables markedly, ranging from 25.1% (*n* = 50) to 81% (*n* = 162) for the specific ASCO/ESMO/GLIM guidelines’ recommendations. Whereas compliance with the guidelines on discussing goals of care, prognosis, treatment options and end-of-life care scored the highest in most variables, most HCPs exhibited low compliance with the discussion about patients’ end-of-life preferences early in the course of incurable illness (49.8%, *n* = 99). There were statistically significant differences between the mean scores of only two variables among the three hospitals in compliance with ASCO/ESMO/GLIM guidelines on the provision

**Conclusion:**

This study indicated that the overall objectively correct knowledge of CC diagnosis criteria was inadequate, with a much lower level of awareness about the ASCO/ESMO/GLIM guidelines on CC and a handful of the HCPs have ever assessed the quality of life of CC patients. Quality improvement interventions on CC diagnosis and management should prioritize improving the level of knowledge on CC, diagnostic criteria and patient-clinician communication, including discussion about patients’ end-of-life care using standardised tools such as ASCO/ESMO or GLIM guidelines on CC using a multidisciplinary team approach.

## Background

More than 50% of people with advanced cancer suffer from cancer-related cachexia (CC) [1]. CC is defined as a multifactorial syndrome characterised by an ongoing loss of skeletal muscle mass (with or without loss of fat mass) that cannot be fully reversed by conventional nutritional support and leads to progressive functional impairment [2]. The pathophysiology is characterised by a negative protein and energy balance driven by a combination of reduced food intake and abnormal metabolism [2], with three phases of clinical relevance; pre-cachexia, cachexia and advanced/refractory [2].

CC is a major contributor to morbidity, associated with progressive impairment in function and quality of life, increased risk of anticancer treatment-related toxicity and with reduced survival [1–3] with devastating effects for patients, their families and health care providers. However, currently, there is no licensed pharmacological treatment or agreed standard of care globally. Disease-modifying therapies and supportive care are the cornerstones of cancer treatment [4]. The American Society of Clinical Oncology (ASCO) and the European Society for Medical Oncology (ESMO) CC guidelines advocate for multimodal interventions for cancer-related cachexia, as a component of supportive care, but note the paucity of approved, effective pharmacological treatment [2–6].

Even though there are some clinical trials being conducted in some countries on CC diagnosis and management, not every CC patient may get included in these trails. Systematic reviews have highlighted that socio-demographic barriers, access to health care, clinical trial inclusion criteria, and attitudes of physicians and patients are factors that limit oncology trial participation [4, 7–11]. Therefore, it is important to improve equity and patient access to CC diagnosis and management, including clinical trials to potentially improve the quality of life for patients living with CC.

The Institute of Medicine (IOM) has recommended *the use of systematically developed guidelines* based on the best available evidence for prevention, diagnosis, treatment and palliative care for cancer patients [12, 13]. The 2021 ASCO presidential address ‘*Equity: Every patient, every day, everywhere’* called for the *urgent need* for equity in access to cancer care [14]. The ASCO, ESMO and the Global Leadership Initiative on Malnutrition (GLIM) [15] guidelines recommend the delivery of CC care utilising a combination of nutrition, physical activity, psychological, oncological, palliative/supportive/rehabilitative care and oncologist competencies [2, 4]. However, *a well-knit combination of these is lacking* in many developing countries, including Uganda.

Also, the current Uganda Cancer Institute Treatment guidelines of 2017 [16] lack content on diagnosis and management of CC, despite the late-stage presentation of most of the cancer patients [12]. Approximately 80% of cancer patients in Uganda are diagnosed at advanced stage [12]. Since CC is the primary cause of death in 22%–30% of cancer patients [1], Uganda may be losing about 22%–30% of cancer patients due to CC. Despite this, there are no specific guidelines and interventions for early diagnosis and management of CC [16] and consequently poor quality of life and survival of cancer patients [12] underscoring *the need for urgent interventions* targeted at early diagnosis and management of CC.

In the June 2022 ASCO meeting, it was noted that** ‘**Advancing Equitable Cancer Care Through Innovation’ is more critical today than ever and noted deep-seated disparities in cancer care globally. Moreover, assessment and management of CC are major challenges for clinicians [2] despite its widespread implications, CC is often poorly diagnosed and often missed completely. Therefore, there is a need for the use of systematically developed guidelines based on the best available evidence for prevention, diagnosis, treatment and palliative care [13].

In line with the recommendations of the ASCO [14, 17]; ESMO [2, 18]; GLIM [19], given the limited information on the treatment options for cancer cachexia [20], and the Clinical Framework for quality improvement (QI) of CC [13], we developed ‘*Applying Science to Strengthen and Improve Systems for Cachexia Care (ASSIST-CC) Study Project in Uganda****.*** The overall goal of this project is to *improve equity* and *access for CC trials* through the application of QI science to alleviate the consequences of cachexia and *improve the quality of life of cachexia patients* in Uganda. In the first phase of this CC research project, the study aimed to assess the level of *awareness and knowledge of CC diagnosis and management and compliance with the ASCO / ESMO/GLIM guidelines on CC* among *healthcare professionals (HCPs) and multidisciplinary teams (MDTs)* involved in the care of cancer patients to improve access to *equitable high-quality cachexia care* for cancer patients.

## Methods and materials

This study was approved by the Uganda Cancer Institute Research and Ethics Committee, approval reference number, UCI-2022-68, and permission to conduct the study was granted by the Uganda National Council for Science and Technology, registration number, HS2656ES.

In this phase one, a total of 200 participants were sampled purposively – multidisciplinary HCPs at the cancer treatment centres or hospital units that provide at least some aspect of cancer care. We included health professionals at the Uganda Cancer Institute, Mbarara Regional Cancer Center & Mbarara Regional Referral Hospital and Kawempe National Referral Hospitals.

A self-administered structured questionnaire developed using the ASCO/ESMO and GLIM guidelines on diagnosis and management of CC was used to assess the level of awareness, and knowledge of the health professionals on CC, and compliance with the ASCO/ESMO/GLIM guidelines on CC-related core communication, barriers to communication, clinician training in communication, discussing goals of care, treatment options and meeting the needs of the underserved populations.

The structured questionnaire spanned the study participants characteristics, what CC is, its diagnosis, management, the core communication skills and tasks that apply across the continuum of cancer care, recommendations that address specific topics, such as discussion of goals of care and prognosis, treatment selection, end-of-life care, facilitating family involvement in care and offer HCPs training in communication skills. A trained study team comprising of nurses, nutritionist, oncologists, social medical worker, public health oncologists and data scientist participated in the data collection and analysis process.

The data were entered into Research Electronic Data Capture software – a secure web-based application for building and managing surveys and databases. All data were analysed using STATA version 18.0 software. The objective knowledge assessment variables were classified as correct response (scored 1) or incorrect response (scored 0). The subjective / perceived knowledge and or awareness variables were classified by a 5-point Likert scale of ‘very low, low, average, high, and very high’ where the study participants answered the questions by stating their level of agreement in five points.

One-way ANOVA, and Kruskal Wallis statistical tests were used after testing for normality and equal variance assumptions to determine whether there were any statistically significant differences between the mean scores of three different groups – Uganda Cancer Institute, Mbarara Regional Cancer Center & Mbarara Regional Referral Hospital, and Kawempe National referral hospitals. One-way ANOVA was applied where the assumption of the dependent variable is normally distributed (gaussian distribution) and there is homoscedasticity (approximately equal variance on the scores across groups). Otherwise, Kruskal-Wallis test was applied in the nonparametric conditions.

## Results

A quantitative survey of self-administered questionnaire was completed by a multi-disciplinary HCPs – physicians, nurses, pharmacists, laboratory scientists/technologists, radiographers/radiologists, medical social workers, public health specialists (including health educators) and other HCPs, including physiotherapists (*N* = 200). Of these participants, nurses and medical doctors constituted the majority (72%) and most (72%) had bachelor and postgraduate degrees (Table 1).

Of all the participants, the overall objectively correct knowledge of CC diagnosis criteria was 67.5% (135), yet there was a much lower level of awareness about ASCO/ESMO/GLIM guidelines on CC at 30% (*n* = 60) and only 21% (*n* = 42) of the HCPs have ever assessed Quality of life of CC patients (Table 2). There were no any statistically significant differences between the mean scores of HCPs Knowledge and awareness of CC & Compliance with ASCO/ESMO/GLIM guidelines among the three hospitals; Uganda Cancer Institute, Mbarara Regional Cancer Center & Mbarara Regional Referral Hospital and Kawempe National referral hospitals (Table 2).

The compliance with ASCO/ESMO/GLIM guidelines on nutritional interventions for patients with CC varied across the variables markedly, ranging from 25.1% (*n* = 50) to 81% (*n* = 162) for the specific ASCO/ESMO/GLIM guidelines’ recommendations (Table 3). There were statistically significant differences between the mean scores of two variables among the three hospitals in compliance with ASCO/ESMO/GLIM guidelines on the provision of additional calories by feeding tubes (*p* = 0.038) and the available evidence to recommend medication to improve CC outcomes (*p* = 0.0286) (Table 3).

Whereas compliance with the ASCO/ESMO/GLIM guidelines on discussing goals of care, prognosis, treatment options and end-of-life cares scored the highest in most variables, most HCPs exhibited low compliance with the discussion about patients’ end-of-life preferences early in the course of incurable illness (49.8%, *n* = 99) (Table 4). Among the three tertiary hospital settings, there was a statistically significant difference between the mean scores of only one variable; clinician’s simplicity of providing information to patients (*p* = 0.0132) regarding discussing goals of care, prognosis, treatment options and end-of-life care (Table 4). There were no statistically significant differences between the mean scores of the HCPs in the other variables on discussing goals of care, prognosis, treatment options and end-of-life care (Table 4).

### Level of knowledge by categories of health-care professionals

**Figure 1.**
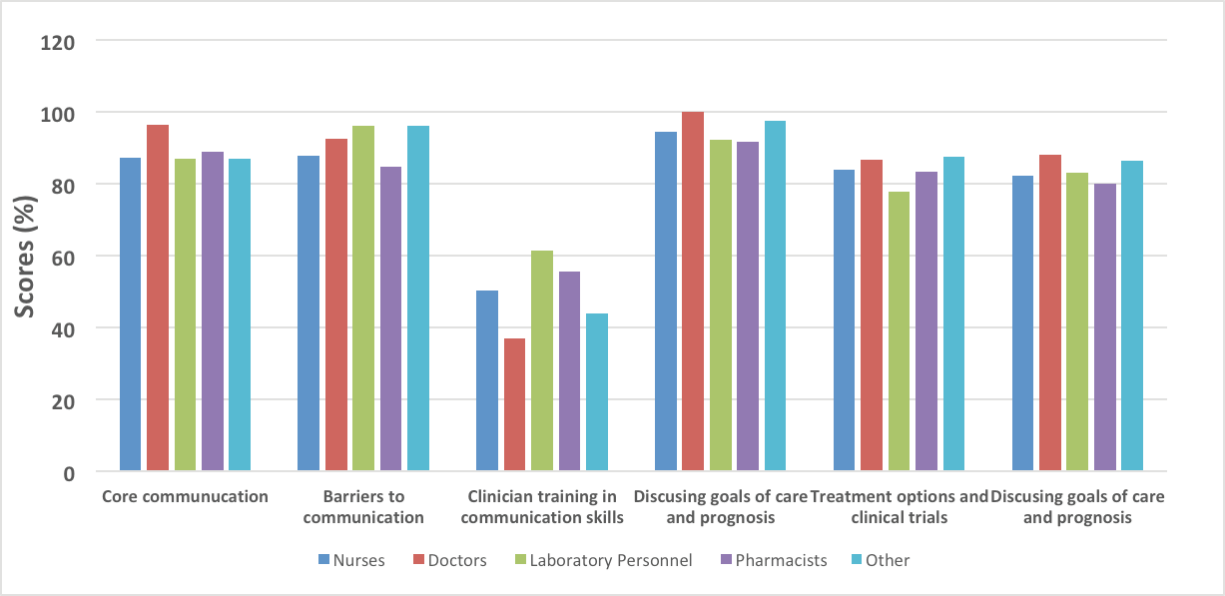
Level of knowledge by categories of health-care professionals.

Further analysis of the level of knowledge on CC, communicating about CC and its management by categories of health-care professionals assessed showed that there were no statistically significant differences among the HCPs (Table 5 and Figure 1).

## Discussion

In the assessment of the level of awareness and knowledge of CC diagnosis and management and compliance with the ASCO/ESMO/GLIM guidelines on CC among HCPs, it was found that the overall objectively correct knowledge score of CC diagnosis criteria was 67.5% (*n* = 135), yet there was a much lower level of awareness about ASCO/ESMO/GLIM guidelines on CC at 30% (*n* = 60). Further analysis of the level of knowledge on CC, communicating about CC and its management by categories of health-care professionals showed that there were no statistically significant differences among the HCPs

Relatively, in an international study conducted throughout Europe, North America and in Japan, only 29.1% of the health professionals recognised a key criterion of CC as weight loss of more than 5% from baseline, yet many did not utilise a standardised definition of CC [21]. Besides, in an online survey of the multi-disciplinary oncology HCPs in Australia and New Zealand, with over 90% of the participants were medical doctors and nurses, 85% of the study participants were not aware of any CC guidelines and 83% considered weight loss ≥10% from the baseline as indicative of CC [22]. Our findings compared with the findings across the world, seem to suggest that the correct understanding of CC diagnosis and management remains a global HCPs challenge, not only in the low- and middle-income countries. This should be viewed and considered as one of the important priority areas for the global oncology research agenda for the current and the next decade.

In addition, in our study, only 21% (*n* = 42) of the HCPs have ever assessed the Quality of life of CC patients. Also, the compliance with ASCO/ESMO/GLIM guidelines on nutritional interventions for patients with CC varied across the variables markedly, ranging from 25.1% (*n* = 50) to 81% (*n* = 162) for the specific ASCO/ESMO/GLIM guidelines’ recommendations. Non-compliance to established guidelines affects the standards of care provided to the patients due to indulgence of personal opinions and varies by professional experience and level of knowledge in the particular disease context. As guided by the IOM, guidelines are derived based on the best available evidence and are systematically developed [23], and thus, HCPs need to comply with them for the provision of better prevention, diagnosis, treatment and palliative care services for cancer patients [12, 13].

Also, whereas compliance with the ASCO/ESMO/GLIM guidelines on discussing goals of care, prognosis, treatment options and end-of-life care scored the highest in most variables, most HCPs exhibited low compliance with the discussion about patients’ end-of-life preferences early in the course of incurable illness (49.8%, *n* = 99). Nevertheless, in our study, on discussing goals of care, prognosis, treatment options and end-of-life care, there was a statistically significant difference between the mean scores of only one variable in the three hospital settings; the clinician’s simplicity of providing information to patients.

Notwithstanding, patients and their family members usually have what they expect from the HCPs and the health system as a whole. In regard to CC, patients are likely to be interested in knowing what could be causing the extreme weight loss and the appropriate interventions for controlling such weight loss. Such information should be provided in plain language, not technical/medical jargon to aid the ease of understanding the health information provided by the HCPs.

Similarly, a study in the UK found that there was a ‘lack of response from HCPs on the management of CC and clarifying to the patients the nature and impact of this syndrome [24]. The UK study indicated that cancer patients and their family members wanted three things from the HCPs: acknowledgement of the weight loss, why profound weight loss, and interventions to deal with it [24]. To address these cancer patient’s needs, HCPs should understand the nature and impact of this syndrome and the current ways of addressing it so that their patients too can understand the nature and impact of CC, especially preventing or controlling the extreme weight loss.

According to the findings from three global surveys on the perspectives of HCPs in14 different countries in North and South America, Asia and Europe, (USA, Mexico, Brazil, Canada, Indonesia, Russia, Turkey, France, Germany, Italy, Poland, Romania, Spain and UK), capacity to promote weight gain was rated as the most important factor for choice of CC treatment with the main goals being ensuring that patients cope with the cancer disease and treatment and improve in their quality of life [25].

Furthermore, in our study, there were statistically significant differences between the mean scores of two important variables among the three hospitals in compliance with ASCO/ESMO/GLIM guidelines on the provision of additional calories by feeding tubes and knowledge on whether there is available evidence to recommend medication to improve CC outcomes, most importantly, avoiding weight loss. Currently, there is a lack of effective and approved pharmacological treatment for CC [4, 5]; however, there are some clinical trials being conducted in some countries.

Moreover, in another nationwide study conducted in 451 designated cancer hospitals across Japan on the perspectives of HCPs on multimodal interventions for CC, in spite of only about half (47.8%) of the HCPs being knowledgeable about the definition of CC guiding them in the diagnosis and management of the syndrome, they tended to consider it important to initiate nutritional and exercise interventions before CC becomes apparent and recognised the importance of holistic multimodal interventions, especially for managing physical and psychological symptoms [26]

The suggested holistic multimodal interventions for CC require interprofessional care, also referred to as MDT. To work well, MDT approach to care of a complex disease or syndrome like CC require investment in education, team practice mentorship and training to improve knowledge and shape positive perception. Interestingly, in Japan, a nationwide electronic survey of multi-disciplinary oncological and general healthcare providers (physicians, nurses, pharmacists, dietitians, rehabilitation therapists and other health professionals) indicated that education, team practice, knowledge and perception on CC management were the main barriers to interprofessional care of CC among the HCPs [27]. Thus, a call for a deliberate institutional effort to nurture team practice, knowledge improvement and building a positive perception of CC syndrome.

To support this, in Australia, a study conducted in a hospital with a dedicated cachexia clinic indicated that improved HCPs’ knowledge and understanding on CC across a staff body can enhance the staff willingness and confidence to address CC and its consequences with patients and their families in a MDT approach [28].

### Strengths and limitations of this study

This is the first study to assess the level of awareness and knowledge of CC diagnosis and management and compliance with the available guidelines on CC among HCPs involved in the care of cancer patients in Uganda. The study participants were multi-disciplinary and clinicians (oncologists and other medical doctors and nurses had the highest levels of participation**.** The participants also had representation of oncology health professionals at the Uganda Cancer Institute – the comprehensive national cancer centre in Uganda, the Mbarara Regional Cancer Center & Mbarara Regional Referral Hospital and Kawempe National referral hospitals. Conversely, the most important limitation of this study is that the findings were based on self-reported data from a self-administered questionnaire, which at times is associated with certain types of response bias, random or systematic. For instance, random response bias may occur if the participant honestly does not know the correct answer to the question but anyway answers the question in spite of reasonably not knowing the answer. Moreover, systematic misreporting could also be conceivable due to cognitive processes, social desirability and the survey environment.

## Conclusion

The baseline findings from this ‘ASSIST-CC showed the overall objectively correct knowledge of CC diagnosis criteria was inadequate, with a much lower level of awareness about the ASCO/ESMO/GLIM guidelines on CC and handful of the HCPs have ever assessed quality of life of CC patients. This study suggests that QI interventions on CC diagnosis and management should prioritize improving the level of knowledge on CC, diagnostic criteria, patient-clinician communication, including discussion about patients’ end-of-life care using standardised tools such as ASCO/ESMO or GLIM guidelines on CC. This should emphasize the involvement of multidisciplinary HCPs nurtured by team practice mentorship and knowledge management for better patient outcomes.

## Conflicts of interest

No conflict of interest exists.

## Funding

This study was funded by Pfizer – Grant No 75383581.

## Consent to participate

All participants provided their written informed consent.

## Consent for publication

Not applicable.

## Research ethical approval

Ethical approval was obtained from the Uganda Cancer Institute Research and Ethics Committee (UCI REC), approval reference number, UCI-2022-68, and permission to conduct the study was granted by the Uganda National Council for Science and Technology (UNCST), registration number, HS2656ES.

## Author contributions

Study concept and design: IA, AJ, JO. Project Management: AJ. Data collection: MN, JS, NN, KN, AA. Data analysis and interpretation JA, AJ. Manuscript writing AJ, JA, IA, SS. Manuscript review and editing: All authors; Final approval of manuscript: All authors.

## Data availability

The dataset generated in this study is not publicly available, but available on reasonable request.

## Figures and Tables

**Table 1.** Social and demographic characteristics of the study participants.

Variable	Variable category	UCI	Mbarara	Kawempe	Overall
		Freq (%)	Freq (%)	Freq (%)	Freq (%)
Age group	Number of participants	142 (17.0)	40 (20.0)	18 (9.0)	200 (100)
	Age (Mean)	36.7	32.9	39.6	36.2
	<30	31 (21.8)	16 (40.0)	2 (11.1)	49 (24.5)
	31–40	86 (60.6)	22 (55.0)	11 (61.1)	119 (59.5)
	41–50	16 (11.3)	2 (5.0)	2 (11.1)	20 (10.0)
	51–60	6 (4.2)	0	3 (16.7)	9 (4.5)
	>60	3 (2.1)	0	0	3 (1.5)
Education level	Certificate	5 (3.5)	1 (2.5)	1 (5.6)	7 (3.5)
	Diploma	31 (21.8)	10 (25.0)	8 (44.4)	49 (24.5)
	Bachelor degree	80 (56.3)	13 (32.5)	8 (44.4)	101 (50.5)
	Postgraduate degree	26 (18.3)	16 (40.0)	1 (5.6)	43 (21.5)
Profession	Nursing	82 (57.8)	18 (45.0)	17 (94.4)	117 (58.5)
	Clinical medicine	16 (11.3)	11 (27.5)	0	27 (13.5)
	Lab technology	19 (13.4)	0	0	19 (9.5)
	Radiography /radiology	2 (1.4)	0	1 (5.6)	3 (1.5)
	Public health	7 (4.9)	1 (2.5)	0	8 (4.0)
	Nutrition	1	0	0	1 (0.5)
	Social work	3 (2.1)	1 (2.5)	0	3 (1.5)
	Pharmacy	9 (6.3)	9 (22.5)	0	18 (9.8)
	Others	4 (2.8)	0	0	4 (2.0)
Religion	Catholic	49 (34.5)	13 (32.5)	13 (72.2)	75 (37.5)
	Anglican	46 (32.4)	14 (35.0)	2 (11.1)	62 (31.0)
	Muslim	13 (9.2)	1 (2.5)	2 (11.1)	16 (8.0)
	Pentecostal	26 (18.3)	10 (25.0)	0	36 (18.0)
	Seventh day adventist	8 (5.6)	2 (5.0)	1 (5.6)	11 (5.5)
Marital status	Single	34 (23.9)	14 (35.0)	2 (11.1)	50 (25.0)
	Married	94 (66.2)	25 (62.5)	14 (77.8)	133 (66.5)
	Separated	8 (5.6)	19 (2.5)	2 (11.1)	11 (5.5)
	Widowed	6 (4.2)	0	0	6 (3.0)

UCI: Uganda Cancer Institute; Mbarara: Mbarara Regional Cancer Center and Mbarara regional referral hospital; and Kawempe: Kawempe National referral hospitals

**Table 2.** Knowledge and awareness of CC & compliance with ASCO/ESMO/GLIM guidelines.

Variable	Responses	Overall	UCI	Mbarara	Kawempe	*p*-value
Knowledge and awareness	Correct (Yes), Incorrect (No)	Freq (%)	Freq (%)	Freq (%)	Freq (%)	
Objective knowledge of CC	Yes	165 (82.5)	114 (80.3)	35 (87.5)	16 (88.9)	0.433
	No	35 (17.5)	28 (19.1)	5 (12.5)	2 (11.1)	
Objective knowledge of CC diagnosis criteria	Yes	135 (67.5)	93 (65.5)	27 (67.5)	15 (83.3)	0.975
	No	65 (32.5)	49 (34.5)	13 (32.5)	3 (16.7)	
Objective knowledge of CC phases	Yes	110 (55.0)	77 (54.2)	22 (55.0)	11 (61.1)	0.366*
	No	90 (45.0)	65 (45.8)	18 (45.0)	7 (38.9)	
Objective knowledge of Sarcopenia	Yes	105 (52.5)	75 (52.8)	21 (52.5)	9 (50.0)	0.572*
	No	95 (47.5)	67 (47.2)	19 (47.5)	9 (50.0)	
Awareness about ASCO/ESMO/GLIM guidelines on CC	Yes	60 (30.0)	46 (32.4)	9 (22.5)	5 (27.8)	0.975*
	No	140 (70.0)	96 (67.6)	31 (77.5)	13 (72.2)	
Ever assessed quality of life of CC patients	Yes	42 (21.0)	33 (23.2)	8 (20.0)	1 (5.6)	0.220
	No	158 (79.0)	109 (76.8)	32 (80.0)	17 (94.4)	
Perceived knowledge of the ASCO guidelines on CC	Very low	71 (35.5)	48 (33.8)	14 (35.0)	9 (50.0)	0.178
	Low	66 (33.0)	50 (35.2)	10 (25.0)	6 (33.3)	
	Average	32 (16.0)	21 (14.8)	8 (20.0)	3 (16.7)	
	High	28 (14.0)	22 (15.5)	6 (15.5)	0	
	Very high	3 (1.5)	1 (0.7)	2 (5.0)	0	
Perceived knowledge of ESMO guidelines on CC	Very low	86 (43.0)	60 (42.3)	17 (42.5)	9 (50.0)	0.702
	Low	60 (30.0)	45 (31.7)	10 (25.0)	5 (27.8)	
	Average	26 (13.0)	16 (11.3)	7 (17.5)	3 (16.7)	
	High	23 (11.5)	18 (12.7)	4 (10.0)	1 (5.6)	
	Very high	5 (2.5)	3 (2.1)	2 (5.0)	0	
Perceived knowledge of GLIM Guidelines on CC	Very low	89 (44.5)	62 (43.7)	17 (42.5)	10 (55.6)	0.447
	Low	64 (32.0)	49 (34.5)	10 (25.0)	5 (27.8)	
	Average	23 (11.5)	11 (7.8)	9 (22.5)	3 (16.7)	
	High	21 (10.5)	18 (12.7)	3 (7.5)	0	
	Very high	3 (1.5)	2 (1.4)	1 (2.5)	0	

* Used one way ANOVA, the rest used Kruskal Wallis

UCI: Uganda Cancer Institute; Mbarara: Mbarara Regional Cancer Center & Mbarara regional referral hospital; and Kawempe: Kawempe National referral hospitals

**Table 3.** Compliance with ASCO/ESMO/GLIM guidelines on nutritional interventions for patients with CC.

Variable/Question	Frequency of correct responses				
	Overall	UCI	Mbarara	Kawempe	*p*-Value
	Freq (%)	Freq (%)	Freq (%)	Freq (%)	
Loss of appetite is common in patients with advanced cancer	56 (28.0)	36 (25.4)	16 (40.0)	4 (22.2)	0.1628
Trying to force a patient to eat increases nausea/vomiting.	146 (73.0)	106 (74.7)	26 (65.0)	14 (77.8)	0.4286
Providing additional calories by feeding tubes does not improve outcomes	50 (25.1)	34 (24.1)	8 (20.0)	8 (44.4)	0.038*
Forcing a patient to eat can affect social interaction with the care taker	130 (65.0)	89 (62.7)	28 (70.0)	13 (72.2)	0.5534
Care givers should give patients other support other than food	132 (66.0)	91 (64.1)	27 (67.5)	14 (77.8)	0.5021
Dietitians may not provide patients with additional opportunities to discuss nutritional challenges	162 (81.0)	114 (80.3)	34 (85.0)	14 (77.8)	0.695*
There is no sufficient evidence to recommend any medication to improve CC outcomes	75 (37.5)	60 (42.3)	13 (32.5)	2 (11.1)	0.0286
Clinicians may offer a short-term trial of a progesterone analog to patients experiencing loss of appetite	131 (65.5)	89 (62.7)	29 (72.5)	13 (72.2)	0.4232
There is no recommendation for other interventions, such as exercise for the management of CC	64 (32.0)	43 (30.3)	14 (35.0)	7 (38.9)	0.6883

* Used one way ANOVA, the rest used Kruskal Wallis

UCI: Uganda Cancer Institute; Mbarara: Mbarara Regional Cancer Center & Mbarara regional referral hospital; and Kawempe: Kawempe National referral hospitals

**Table 4.** Discussing goals of care, prognosis, treatment options and end-of-life care.

Domain	Variable/Question	Frequency of correct responses				
		Overall	UCI	Mbarara	Kawempe	*p*-value*
		Freq (%)	Freq (%)	Freq (%)	Freq (%)	
Discussing goals of care and prognosis	Clinicians should provide diagnostic and prognostic information that is tailored to the patient’s needs	185 (92.5)	130 (91.6)	39 (97.5)	16 (88.9)	0.3762
	Clinicians should reassess a patient’s goals, priorities and desire for information	192 (96.0)	136 (95.8)	39 (97.5)	17 (94.4)	0.8333
	Clinicians should provide information in simple and direct terms	196 (98.0)	140 (98.6)	40 (100)	16 (88.9)	0.0132
	When providing bad news, clinicians should take additional steps to address the needs and responses of patients.	187 (94.0)	131 (92.9)	40 (100)	16 (88.9)	0.1613
Discussing treatment options and clinical trials	Clinicians should clarify the goals of treatment so that the patient understands likely outcomes and can relate the goals of treatment to their goals of care	189 (94.5)	132 (93.0)	40 (100)	17 (94.4)	0.2273
	Clinicians should provide information about the potential benefits and burdens of any treatment and check the patient's understanding of these benefits and burdens.	191 (95.5)	133 (93.7)	40 (100)	18 (100)	0.1473
	Clinicians should discuss treatment options in a way that preserves patient hope, promotes autonomy and facilitates understanding.	171 (85.5)	123 (86.6)	123 (86.6)	14 (77.8)	0.6028
	Clinicians should not make patients aware of all treatment options, when appropriate, clinicians should discuss the option of initiating palliative care simultaneously with other treatment modalities.	135 (67.5)	99 (69.7)	24 (60.0)	12 (66.7)	0.5109
	If clinical trials are available, clinicians should start treatment discussions with standard treatments available off trial		106 (74.7)	35 (87.5)	12 (66.7)	0.1414
Discussing end of life care	Clinicians should use an organised framework to guide the bidirectional communication about end-of-life care with patients and families.		133 (93.7)	37 (92.5)	15 (83.3)	0.2946
	Clinicians should not initiate conversations about patients’ end-of-life preferences early in the course of incurable illness		77 (54.2)	15 (38.5)	7 (38.9)	0.1384
	Clinicians should explore how a patient’s culture, religion, or spiritual belief system affects their end-of-life decision making		129 (90.9)	38 (95.0)	16 (88.9)	0.6499
	Clinicians should refer patients and families to psychosocial team members		132 (93.0)	40 (100.0)	18 (100)	0.1178
	Clinicians should suggest family in discussions early in the course of the illness for support and discussion about goals of care.		125 (88.0)	36 (90.0)	15 (83.3)	0.771

* *p*-value based on Kruskal Wallis statistical test

UCI: Uganda Cancer Institute; Mbarara: Mbarara Regional Cancer Center & Mbarara regional referral hospital; and Kawempe: Kawempe National referral hospitals

**Table 5.** Level of knowledge by categories of health-care professionals.

	Nurses (*n* = 117)	Doctors (*n* = 27)	Laboratory personnel (*n* = 19)	Pharmacists (*n* = 18)	Others (*n* = 19)	p-value
Domain	%	%	%	%	%	%
Core communication	87.2	96.3	86.8	88.9	86.8	0.355
Barriers to communication	87.8	92.6	96.1	84.7	96.1	0.413
Clinician training in communication skills	50.1	37.0	61.4	55.6	43.9	0.423
Discussing goals of care and prognosis	94.4	100	92.1	91.7	97.4	0.399
Treatment options and clinical trials	83.8	86.7	77.9	83.3	87.4	0.491
Discussing goals of care and prognosis	82.2	88.1	83.2	80.0	86.3	0.610
